# PlasmidHawk improves lab of origin prediction of engineered plasmids using sequence alignment

**DOI:** 10.1038/s41467-021-21180-w

**Published:** 2021-02-26

**Authors:** Qi Wang, Bryce Kille, Tian Rui Liu, R. A. Leo Elworth, Todd J. Treangen

**Affiliations:** 1 Systems, Synthetic, and Physical Biology (SSPB) Graduate Program, Rice University, Houston, Texas 77005 USA; 2 Department of Computer Science, Rice University, Houston, Texas 77005 United States

**Keywords:** Synthetic biology, Genome informatics, Software

## Abstract

With advances in synthetic biology and genome engineering comes a heightened awareness of potential misuse related to biosafety concerns. A recent study employed machine learning to identify the lab-of-origin of DNA sequences to help mitigate some of these concerns. Despite their promising results, this deep learning based approach had limited accuracy, was computationally expensive to train, and wasn’t able to provide the precise features that were used in its predictions. To address these shortcomings, we developed PlasmidHawk for lab-of-origin prediction. Compared to a machine learning approach, PlasmidHawk has higher prediction accuracy; PlasmidHawk can successfully predict unknown sequences’ depositing labs 76% of the time and 85% of the time the correct lab is in the top 10 candidates. In addition, PlasmidHawk can precisely single out the signature sub-sequences that are responsible for the lab-of-origin detection. In summary, PlasmidHawk represents an explainable and accurate tool for lab-of-origin prediction of synthetic plasmid sequences. PlasmidHawk is available at https://gitlab.com/treangenlab/plasmidhawk.git.

## Abstract (web summary)

Advances in synthetic biology and genome engineering raise awareness of potential misuse. Here, the authors present PlasmidHawk, a sequence alignment based method for lab-of-origin prediction.

## Introduction

Thanks to the advancement of genome engineering and sequencing technology, researchers now have the capability to readily read and write DNA sequences^1^. This new technology has the promise of significantly improving the quality of human life through various fields, such as increasing agricultural yields^2^, accelerating drug discovery^3^ or advancing gene therapy^4^. While the use cases of this exciting technology enabling the bio-economy are largely positive, biosecurity, IP infringement, and potential misuse remain as concerns^5,6^. As a proof of principle, in response to previously outlined concerns, Allen et al. utilized a set of signature sequences with length *k*, also referred as signature *k*-mers, to differentiate artificial sequences from natural genomes and plasmids^7^. Although this promising *k*-mer-based matching approach offers the ability to distinguish artificial sequences from a set of background sequences, there is still a need to develop a predictive pipeline that enables the handling of an enormous amount of input sequences and reveals finer details of a given synthetic sequence. To meet the need, Nielsen et al. introduced a software tool to link artificial sequences with their depositing labs by using deep learning^8^. Despite the complex computational challenge, the prediction accuracy was promising: 48% accuracy in correctly identifying the lab-of-origin for an unknown sequence if allowed one prediction, and up to 70% accuracy if allowed ten predictions. To date, deep learning has been wildly applied in analyzing genomic data as the amount of data has grown larger and more complex^9^. Applications of deep learning include gene annotation^10^, sequence pattern identification^11^, discovering biomarkers^12^, and inferring cell function and structure^13^. At its core, machine learning, and in particular deep learning, is utilized for classification based on training data and learning hidden patterns and structure in the data^14^. Although deep learning-based approaches have been at the core of tremendous successes and popularity in many areas, including computer vision^15^, natural language processing^16^, and robotics^17^, it has some intrinsic disadvantages. First, it has limited explainability; models are often unable to fully detail the features and decision-making process that led to a given classification or prediction^18^. Second, the computational cost and carbon footprint of such methods are skyrocketing while processing ever-increasing amounts of biological data^19^. Third, the predictions heavily rely on representative training data and the choice of hyperparameters^20^.

To address this, we introduce a fully transparent, efficient, explainable approach to assist end users in identifying the lab-of-origin of engineered DNA sequences. We solve the synthetic sequence tracking problem from an alignment perspective. Specifically, we make predictions by integrating the information of common sequences and “signature” sequences used in plasmid constructs via a pan-genome data structure. Pan-genomes have been studied for nearly two decades^21^. Pan genomes serve as a high-level summary of a biologically related group of genomes by capturing all of the group’s core and accessory regions, though the exact definition of a pan-genome can vary based on the application. In this paper, we define a pan-genome as a collection of genomic regions that are common or unique to synthetic biology research labs. Pan-genomes are frequently applied to capture the genomic diversity of a bacterial clade^22^. Many bioinformatic tools have been developed to build pan-genomes, such as Roary^23^, seq-seq-pan^24^, VG^25^, and Plaster^26^. Plaster offers a linear time construction algorithm enabling it to scale to massive DNA sequence repositories. Building off of our prior work, we have developed a pan-genome for all available synthetic plasmid sequences using Plaster. We then use this synthetic sequence pan-genome as a framework for predicting the lab-of-origin of previously unseen, newly engineered sequences.

In this study, we demonstrate that pan-genome alignment combined with a lab score correction technique can successfully predict the lab-of-origin of an engineered DNA sequence 75.8% of the time. Around 85.2% of the time the source lab is included in the top 10 predicted labs. This approach has very few pre-processing steps, a quick update time for adding new known sequences, and a detailed and interpretable explanation for its predictions. This is in stark contrast to the previous convolutional neural network (CNN) model which must be retrained to incorporate a single newly labeled sequence, and which is intrinsically a black box model.

## Results

### Neural network vs. PlasmidHawk performance

**Fig. 1:**
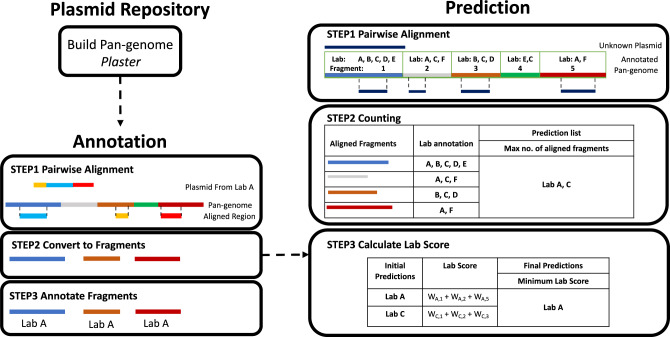
PlasmidHawk Pipeline. First, a pan-genome from the Addgene plasmids is built by Plaster. The plasmids are then aligned back to the final pan-genome to annotate the pan-genome with the depositing lab information for each aligned fragment. To predict the lab-of-origin of an unknown plasmid, PlasmidHawk aligns the unknown plasmid to the annotated pan-genome (Prediction Step 1) and counts the number of aligned fragments for each lab (Prediction Step 2). PlasmidHawk returns the labs that have the maximum number of aligned fragments as the predicted labs for lab-of-origin (Prediction Step 2). Finally, PlasmidHawk calculates the plasmid lab score for each lab. This step takes the labs with the minimum lab score as a final refinement step of its predictions for lab-of-origin (Prediction Step 3).

We have developed a software called PlasmidHawk to predict the lab-of-origin of unknown synthetic DNA sequences. Lab-of-origin prediction with PlasmidHawk consists of three steps. The first step is to build a pan-genome from the synthetic plasmid training data using Plaster^26^. Second, in addition to building the pan-genome, PlasmidHawk annotates the pan-genome with records of which unique plasmid regions originated from which depositing labs. Lastly, PlasmidHawk predicts the lab-of-origin of new, unseen plasmids in the test data set by aligning those plasmids to the previously constructed and annotated pan-genome (Fig. 1).

To begin the experiments, we select full plasmid sequences from labs who have at least ten deposited plasmids in Addgene for use as our input dataset. There are a total of 38,682 full-length plasmids from 896 labs. We split the sequences into two groups: the training group and the testing group. We use the training sequences to construct and annotate the synthetic plasmid pan-genome sequence *P*_train_. The pan-genome *P*_train_ contains a set of unique sequence fragments. Each fragment is stored as an independent entry in a multi-fasta file. Fragments in the fasta file form a linear pan-genome. Each fragment is further annotated with a list of the depositing labs who have sequences that align to the fragment. After building and annotating the pan-genome, we predict the lab-of-origin of the sequences in the test set. In order to identify the lab-of-origin of an unknown plasmid, PlasmidHawk aligns a given test plasmid *p* to the input pan-genome and identifies aligned regions in the pan-genome. While aligning to the pan-genome, the test sequences are broken into a set of sub-sequences. In this study, we also refer to the test sequence as a query sequence. Each sub-sequence from the query sequence can potentially align to multiple fragments in the pan-genome. These sub-sequences can also overlap with each other inside of the query sequence. (Fig. 1 Prediction Step 1). It then selects the pan-genome fragments that overlap with those aligned regions. We refer to these selected fragments as aligned fragments for the plasmid *p*. After identifying the aligned fragments, PlasmidHawk uses the fragments to predict the depositing lab or labs. Though PlasmidHawk, for instance in MAX mode, can return multiple labs as the most likely labs of origin, for this study we only allow one lab to be the true depositing lab. PlasmidHawk has two prediction modes: MAX mode and CORRECT mode. MAX mode predicts the lab-of-origin based on the set of labs who have the maximum number of aligned fragments for the plasmid *p* (Fig. 1 Prediction Step 2). Alternatively, CORRECT mode returns the lab or labs with the minimum “lab score”. CORRECT mode attempts to refine the set of labs from MAX mode by calculating the lab score which we introduce in this work. The lab score is calculated by weighting aligned fragments for each lab (Fig. 1 Prediction Step 3) (see the “Methods” section). In our experiments, all depositing labs are always present inside of the training data for both PlasmidHawk and the CNN benchmark experiments. In the real world, however, this may not always be the case. In the case where the true depositing lab has no presence in the training data, a query sequence can still align to the pan-genome, and PlasmidHawk will return the best matching of the previously seen labs. By the definitions used in our evaluations, this would effectively be a false negative. That is, the true lab-of-origin would not be in the top-N labs predicted by PlasmidHawk. It is worth noting that machine learning methods are also affected by labs not in the training dataset. Despite this, as shown in Supplementary Note 1, PlasmidHawk can still provide useful predictions by pinpointing labs that are closely related to the true depositing lab’s plasmid.

**Fig. 2:**
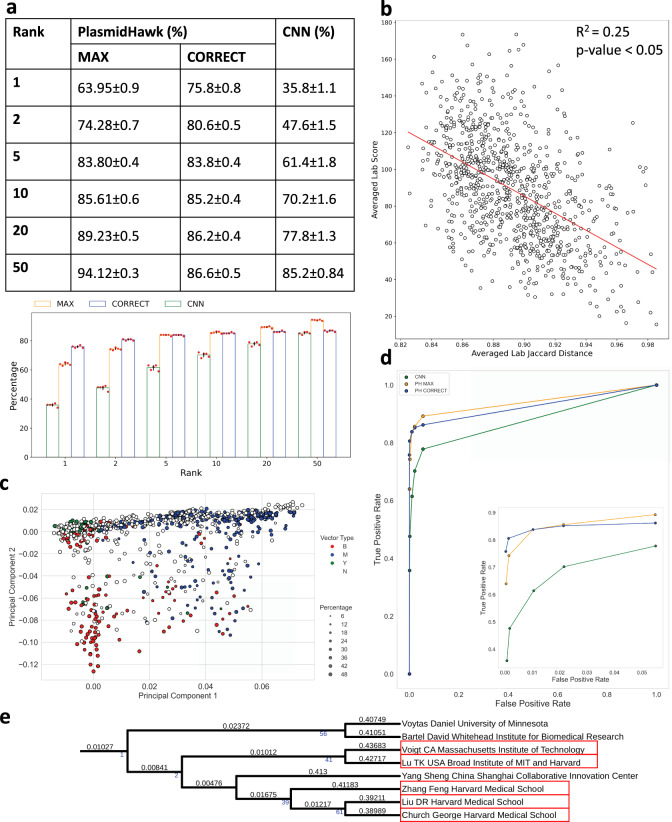
Prediction results and statistical analysis. **a** The performances of plasmid lab-of-origin prediction using PlasmidHawk and the CNN. Data represents mean ± SD of *N* = 5 independent experiments. **b** Linear regression analysis between averaged lab Jaccard distances and averaged lab scores. Each dot represents a lab. The *x* axis shows averaged lab Jaccard distances. The larger the averaged lab Jaccard distance is, the more unique a lab’s plasmids are. The *y* axis is the averaged lab score. Labs with smaller averaged lab scores are more likely to be returned by PlasmidHawk CORRECT mode as predicted source labs. Two-sided *p*-value is calculated (*p* = 3.23*e*−56). **c** Principal component analysis of labs based on lab Jaccard distances. The colors label labs based on their sequences’ host cells. The size of the dot corresponds to the percentage of the most abundant host cells inside a lab. Blue: mammalian lab (M), Green: yeast lab (Y), Red: bacterial lab (B), black: N/A lab (NA). **d** Receiver operating characteristic (ROC) curve for the CNN and PlasmidHawk. **e** A clade of the lab-relatedness tree. Branch lengths are shown on the top of the branches. Support values are annotated under the branches and marked in blue. Support values equal to 0 are not displayed. Labs who belong to the same academic family tree or have collaborated with each other are highlighted in red.

To evaluate the performance of PlasmidHawk, we reran the deep learning experiments based on the description in Nielsen et al. ^8^. We used the same data set used for the PlasmidHawk lab-of-origin prediction experiments. We construct and train the same CNN to predict the lab-of-origin of synthetic sequences. The final trained CNN can predict the correct lab-of-origin 35.8% of the time. The correct lab is ranked within the top 10 predicted labs 70.2% of the time (Fig. 2a). Our CNN prediction results are only slightly lower than the reported results in Nielsen et al., in which the accuracy is 48%, and 70% of the time the depositing lab is included in the top 10 predicted labs. Therefore, we believe our CNN experiments can fairly represent the performance of CNNs in lab-of-origin predictions. We believe the cause for the slight drop in performance between our CNN and the CNN built in Nielsen et al. is the larger size of our more up-to-date training dataset and the minor difference in how the true depositing source labs for each sequence were determined (see the “Methods” section). We can further optimize the neural network to improve the results, but, in general, we do not expect a significant boost in prediction accuracy.

To begin comparing the lab-of-origin prediction accuracy between the CNN and PlasmidHawk MAX mode, we only consider the PlasmidHawk MAX mode prediction results, considering only the case where the true source lab is reported as the single best prediction. A prediction result containing more than just the source lab in the highest scoring prediction set, or that does not contain the true source lab in this set, is classified as an incorrect prediction. In this case, PlasmidHawk MAX mode can reach 63.95% accuracy. To further compare the results between the CNN and PlasmidHawk MAX mode, we calculate the accuracies when considering the 2, 5, 10, 20, and 50 best scoring predicted labs (Fig. 2a) (see the “Methods” section). Overall, PlasmidHawk MAX mode outperforms the CNN with respect to both TPR and FPR (ROC curve in Fig. 2d).

PlasmidHawk CORRECT mode identifies source labs by calculating lab scores for all the labs selected from MAX mode (see the “Methods” section). The lab(s) with the lowest lab score values are considered as the final lab-of-origin predictions. Using the same test data sets used in the MAX mode prediction experiments, PlasmidHawk CORRECT mode has around 75.8% accuracy. 85.2% of the time the source lab is in the top 10 predicted labs (Fig. 2a). In addition, when compared to the CNN approach, the accuracies of both MAX mode and CORRECT mode have lower standard deviations (Fig. 2a). This means that in general lab-of-origin predictions from PlasmidHawk are more consistent.

### PlasmidHawk prediction and confidence

The number of labs returned by PlasmidHawk MAX mode and the ranking of labs in CORRECT mode reflect the confidence PlasmidHawk has in its lab-of-origin prediction. If MAX mode outputs only one lab as the potential depositing lab, around 92% of these predictions are right. If MAX mode outputs two labs, roughly 66% of the time CORRECT mode is able to distinguish the right depositing lab from those two candidates (Supplementary Fig. 1a). As the number of labs predicted by MAX mode increases, the accuracy will decrease, though it remains better than random guessing. In summary, the fewer labs returned by MAX mode, the more confident PlasmidHawk’s final lab prediction is.

Similarly, the ranking of predicted labs in the PlasmidHawk CORRECT mode is also associated with the confidence PlasmidHawk has in its prediction. Supplementary Fig. 1b shows the plasmids, whose depositing labs are returned by PlasmidHawk MAX mode (the number of labs returned by MAX mode is larger than 1) and then ranked by CORRECT mode. In around 54% of these cases, the top 1 plasmid ranked by CORRECT mode will be the correct source lab. As the true lab ranks lower in the CORRECT mode, the probability that the lab is the true source lab is lower. Therefore, the ranking of predicted labs in CORRECT mode is also a representation of PlasmidHawk’s confidence level. The higher the rank, the higher confidence PlasmidHawk has that it is the likely lab-of-origin.

### Lab scores and lab sequence similarities

PlasmidHawk CORRECT mode lab-of-origin experiments show that calculating the lab score can significantly improve the accuracy of predicting source labs (Fig. 2a). Lab scores are calculated by summarizing all the weights of different parts of query sequences. The magnitude of the weights are decided by the uniqueness of the sequences (see the “Methods” section). PlasmidHawk then normalizes the lab scores and chooses labs with the minimum lab score as the final lab-of-origin prediction. Based on the workflow of CORRECT mode, we posit that labs with more unique sequences are more likely to have lower lab scores, that is, be identified as depositing labs.

To validate our hypothesis, we propose to describe the relationship between lab scores and the uniqueness of each lab using a regression model. First, we need to quantify the dissimilarities of a lab’s plasmids among other labs. To do that, we employ Jaccard distances, also called lab Jaccard distances in this paper, to estimate the differences between two labs’ sequences (see the “Methods” section). A large pairwise lab Jaccard distance indicates there are few shared sequences between two labs. To summarize the idiosyncrasies of a lab’s sequences, we average all the pairwise lab Jaccard distances for individual labs, in order to generate a single value, referred to as the averaged lab Jaccard distance, to represent the uniqueness of a lab’s sequences compared to the other 895 labs’ sequences. Labs with a large number of plasmids (more than 200 plasmids) are likely to have large averaged lab Jaccard distances. However, the majority of labs (labs with <200 plasmids) have no obvious relationship between the number of sequences each lab has and the averaged Jaccard distances (Supplementary Fig. 2). Therefore, it is reasonable to use the averaged lab Jaccard distances to represent the number of unique sequences a lab has, as the value of the averaged lab Jaccard distances is not heavily impacted by the number of plasmids a lab has. A lab with few unique sequences tends to have small averaged lab Jaccard distances. Additionally, we have further explored lab sequence diversity by introducing between-lab and within-lab research diversity scores (Supplementary Note 2). To understand the relationship between averaged lab Jaccard distances and PlasmidHawk predictions, we calculate averaged lab scores for each source lab (see the “Methods” section). In general, the smaller averaged lab score a lab has, the more likely the lab is nominated as the true source lab by PlasmidHawk CORRECT mode.

After getting averaged lab scores and averaged Jaccard distances for all the depositing labs, we fit a linear regression model between these two variables. As averaged lab Jaccard distances increase, averaged lab scores decrease (*P* value < 0.05, *R*^2^ = 0.25) (Fig. 2b). The result shows that lab scores reflect the distinctness of labs sequences. It also indicates that CORRECT mode tends to link query sequences with labs who have more unique sequences.

### Detailed comparison between neural network and PlasmidHawk predictions

In this section, we examine the results from the five benchmark experiments in detail. In the five experiments, the testing plasmids (with replacement) from 183 labs were never correctly identified by the CNN (when outputting only the top 1 predicted lab). In contrast, testing plasmids from only seven labs were never successfully identified by PlasmidHawk. Among these seven labs, four of them were also not correctly predicted by the CNN. The four labs are: Root David, Ferrer J, Baltimore David, and Gunawardane RN labs.

In order to better characterize what kind of plasmids are difficult to identify for lab-of-origin prediction, we look at plasmids from labs that have never been successfully detected by both the CNN and PlasmidHawk. The David Root lab has the most plasmids in the entire dataset with a total 2717 plasmids, so, theoretically, it should be the easiest one to be identified as there are many records of plasmids from this lab in the training set. However, neither the CNN nor PlasmidHawk can correctly pinpoint David Root plasmids. Therefore, we selected this lab for an in depth case study. Among those 2717 plasmids, 2696 plasmids are from Doench et al. paper and created for optimizing sgRNA^27^. They were constructed using the Addgene plasmid 52,963 as the main backbone and introduced 1-nucleotide mutations to the sequence. This example demonstrates that plasmids with single nucleotide polymorphisms (SNPs), instead of large signature sequences, are hard to correctly identify the lab-of-origin for both PlasmidHawk and the CNN approach.

Additionally, we examined the individual plasmids to see which plasmids are correctly predicted only by the CNN, but not by PlasmidHawk. Among all the plasmids predicted correctly by the CNN, the majority of them are also successfully predicted by PlasmidHawk. However, 626 plasmids’ lab-of-origin fail to be identified by PlasmidHawk (Supplementary Fig. 3a). Several reasons can explain why some plasmids are correctly predicted by the CNN, but not by PlasmidHawk. First, we examine the sequences from labs whose plasmids are only ever correctly predicted by the CNN but not by PlasmidHawk. Three labs satisfy this criteria: Winslow MM, Omura M, and MPI CBG PEPC labs. From the Omura M lab we can see one difficulty for PlasmidHawk. Testing plasmids from Omura M are either mistaken as having been deposited by the Moravec Tomáš or the Baylies MK lab. Based on the lab Jaccard distances, Moravec Tomáš (JD:0.876) and Baylies MK (JD:0.878) are the most similar labs to Omura M. This example demonstrates that PlasmidHawk can have trouble distinguishing plasmids from labs with similar plasmids.

The second explanation is that, based on the formula of the lab score, labs with large numbers of fragments are less likely to be selected by PlasmidHawk CORRECT mode (Supplementary Fig. 3b). When multiple labs are selected by PlasmidHawk MAX mode and they all align to the same (or similar) fragments, PlasmidHawk CORRECT mode tends to pick labs with fewer fragments. This principle, which PlasmidHawk CORRECT mode is built on, can be successfully applied to most situations. As labs with fewer fragments are less likely to have the same or similar set of aligned fragments by chance, labs with fewer fragments have a higher chance to be the true depositing labs. However, this hypothesis does not apply to all scenarios. Because of this, we are able to observe cases where labs with large numbers of fragments are predicted correctly by the CNN but not by PlasmidHawk.

Next, we wanted to look into the performance of PlasmidHawk vs. the CNN approach across a range of averaged lab Jaccard distances. We find that from averaged lab Jaccard distances of 0.82–0.96 the prediction accuracy steadily increases for both methods, with PlasmidHawk accuracy higher than the CNN approach (Supplementary Fig. S4). When the averaged lab Jaccard distances increase above 0.96, the accuracy of PlasmidHawk decreases slightly while the CNN accuracy increases substantially. Labs with large averaged lab Jaccard distances (0.98–1.0) usually have large numbers of pan-genome fragments (Supplementary Fig. S5). If two labs have the same set of aligned fragments for a query sequence, the lab with more pan-genome fragments is less likely to be picked by PlasmidHawk based on the lab score equation, offering an explanation for this trend.

### Lab clustering and lab-relatedness tree

DNA engineering techniques have revolutionized the way people study biology. Among them, engineered plasmids are widely used as vehicles to deliver and express non-native DNA into different host cells. At the beginning, scientists focused on expressing engineered plasmids inside bacteria. Recently, as DNA manipulation techniques have matured, people have shifted their attention from bacterial to mammalian cells in order to control and understand more complex cellular systems. Despite the rapid development in synthetic biology, there is no suitable approach to compare sequence similarities across different labs. The averaged lab Jaccard distance provides an initial attempt in comparing the uniqueness of individual labs’ sequences. In addition, the averaged lab Jaccard distance provides a way to quantify the development of plasmid engineering. Since one of the major trends in plasmid engineering is the shift from bacterial to mammalian hosts, in this section we evaluate the uniqueness of a lab’s sequences from a host cell perspective.

To do that, we first classify labs into different groups based on the highest percentage of their plasmids’ host cells. To simplify this problem, even though there are many different types of vector hosts shown in Addgene, we simply classify labs into four categories: mammalian (M), bacterial (B), yeast (Y), or N/A (NA). If a lab has more mammalian vectors than other types of vectors, the lab will be labeled as a mammalian lab (M). If a lab has more plasmids belonging to a type other than these three types of vectors, it is classified as N/A.

To identify the distribution of different target host cells across labs, we label their lab type in Fig. 2b to generate Supplementary Fig. 5. The size of each dot corresponds to the percentage of the prominent vector hosts for each lab (ties are classified as N/A). Supplementary Fig. 5 shows that labs focusing on engineering mammalian vectors are likely to have lower averaged lab Jaccard distances and higher averaged lab scores. Based on this result, we may roughly conclude that synthetic plasmids expressed in mammalian cells have lower sequence diversities and are less prone to have their lab-of-origin be identified by PlasmidHawk CORRECT mode than plasmids designed for yeast and bacterial cells.

In addition, we generated a PCA plot using the distance matrix of lab Jaccard distances for all labs and color the labs based on their host classification (Fig. 2c). The PCA plot shows a clear separation between mammalian labs and bacterial labs along PC1. PC1 recovers the variation of host vector lab types and reveals these lab types as distinct, visually apparent clusters spanning across PC1. PC2 captures the variation within lab type group clusters. The principal component analysis further verifies our findings in Supplementary Fig. 5.

Furthermore, we construct a lab-relatedness tree using lab Jaccard distances to reveal the academic relationships among all the labs (see the “Methods” section). Figure 2e displays one of the clades of the lab-relatedness tree. Branch lengths represent the distances between labs. In Fig. 2e, principal investigators who belong to the same academic family or have collaborated with each other are highlighted by the same color. A collaborative relationship is confirmed if two labs have joint publications, patents, and/or research projects (Supplementary Table 4). Some labs, such as the Yang Sheng Lab, appear in the same clade without any validated collaborations with other labs. Those labs are grouped together due to their plasmids similarities (e.g. CRISPR vectors), which implies that their research fields are closely related. The lab-relatedness tree, which is derived from the alignment between the synthetic plasmid pan-genome and the original plasmid sequences, has the potential to reveal the academic genealogies in addition to being used for bioforensics.

### Comparisons with BLAST-based and CNN-based approaches

In Nielsen et al., researchers hand selected a plasmid from the Voigt lab (pCI-YFP,JQ394803.1) which exists in Genbank but not in the Addgene dataset, to compare the performances of the CNN and BLAST in identifying the source lab for an unknown sequence. BLAST fails to predict the lab-of-origin of pCI-YFP, ranking the Voigt lab 5th in its prediction. On the other hand, the CNN approach correctly predicted the plasmid with a significant *p*-value.

**Fig. 3:**
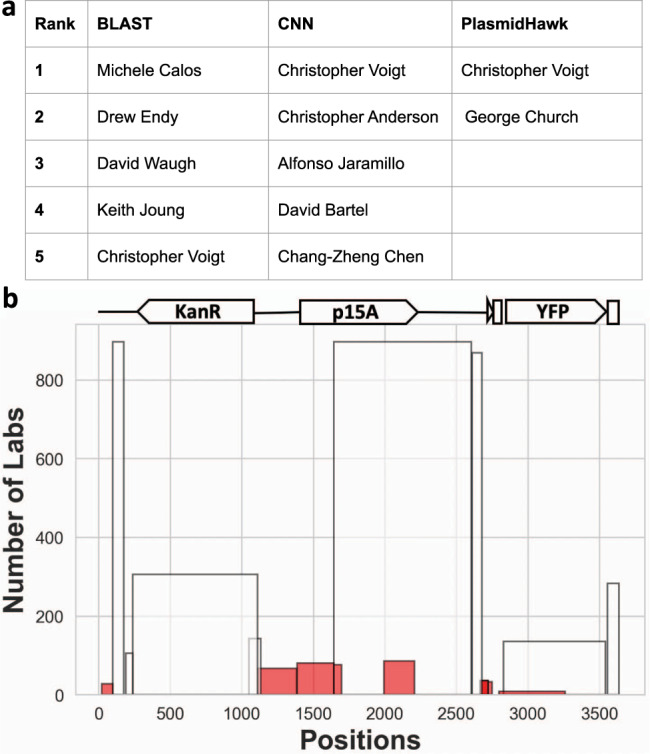
BLAST, CNN, and PlasmidHawk prediction comparisons and interpretation. **a** BLAST, CNN, and PlasmidHawk lab-of-origin prediction results. **b** The number of labs annotated for the regions of pCI-YFP that aligned to different fragments in the synthetic pan-genome. Each bar represents a fragment aligning to the positions in pCI-YFP. The height of the bar represents the number of labs annotated with that fragment. Pan-genome fragments with <100 labs annotated with have aligned to pCI-YFP at 21–98nt, 1110–1697nt, 1993–2209nt, 2667–2751nt and 2797–3260nt positions (red bars). For clarity, the figure only displays a subset of the aligned fragments. The complete set of aligned fragments are shown in Supplementary Fig. 6.

To evaluate PlasmidHawk’s performance, we predict the source lab for pCI-YFP using PlasmidHawk. We input the complete pan-genome *P*_c_, which compacts all the plasmids from labs who have at least 10 deposited full sequences in Addgene. Two labs, including George Church and Christopher Voigt, are identified as the set of top lab predictions using PlasmidHawk MAX mode. PlasmidHawk CORRECT mode further identifies Christopher Voigt as the true depositing lab (Fig. 3a).

One of the advantages of using PlasmidHawk is that it can provide the alignment details of a given plasmid to explain the reasoning behind its prediction (Fig. 3b). pCI-YFP aligns to a total of 54 fragments in *P*_c_. The two labs selected by PlasmidHawk have 21 out of 54 fragments. Among those 21 fragments in Voigt lab, 11 of them have been used by more than 100 labs (white bars in Fig. 3b). Those are common sequences used in the plasmid construction process. 21–98nt, 1110–1697nt, 1993–2209nt, 2667–2751nt, and 2797–3260nt in pCI-YFP are signature regions that help PlasmidHawk narrow down the number of lab candidates (red bars in Fig. 3b). All of these regions, except 2797–3260nt, have been identified in Nielsen et al. as well using deep learning disruption analysis. For the subsequence around 2797–3260nt, PlasmidHawk identifies a pan-genome fragment, which is shared by only eight labs (including Voigt lab), mapping to that region in pCI-YFP. Notably, the importance of this 463nt region does not stand out in the deep learning disruption analysis.

Furthermore, the PlasmidHawk alignment results show that subsequences at insertion sites (21–98nt, 1110–1697nt, 2667–2751nt and 2797–3260nt) and rarely used genetic parts (p15A, 1993–2209nt) are crucial in lab-of-origin detection. Specifically, fragments aligned to common gene parts (e.g. KanR and YFP) alone are annotated with lots of labs. However, fragments that cover both the genetic elements and the backbone sequences (1110–1697nt and 2797–3260nt) are shared by few labs. While inserting genes into a backbone, researchers usually inevitably leave signatures on the plasmids. Those “scar” sequences help us to identify the true depositing labs. Additionally, some gene parts, which are not commonly used, can also serve as potential signatures. For example, although the origin of replication is essential in plasmid construction, the p15A replication origin (1993–2209nt) has only been used by <100 labs. Therefore, p15A can potentially serve as an important lab-of-origin detection signal.

### Function of signature sequences

To further understand the techniques that are readily traceable, we examined the function of the signature sequences identified by PlasmidHawk. As 57% of correctly predicted plasmids have aligned fragments with only their depositing lab annotated (Supplementary Fig. 7), we considered aligned fragments from those successfully identified testing plasmids, which have only the annotation of the true depositing lab, as signature sequences. We then used Prokka to report a functional annotation for the signature sequences^28^.

In the five experiments, a total of 5817 fragments were selected as signature sequences. Among them, 2008 fragments are predicted as hypothetical proteins. Excluding the hypothetical proteins, serine/threonine-protein kinase PknD appears most frequently (17 times). Thus, serine/threonine-protein kinase PknD is potentially the most easily traceable genetic element. In addition, CRISPR-associated endonuclease proteins have been used as signature sequences multiple times (Cas1, Cas2, and Cas9) (Supplementary Fig. 8).

## Discussion

This study demonstrates that aligning unknown sequences to the synthetic plasmid pan-genome can effectively identify engineered DNA sequences’ lab-of-origin. PlasmidHawk achieves better accuracy than state-of-the-art neural network-based approaches while providing interpretable prediction results, identifying unique sequence fragments responsible for identifying the source labs. As shown in the case of pCI-YFP, PlasmidHawk can even help elucidate a more in depth story of shared sequence fragments that are shared by many labs when constructing their synthetic plasmids as opposed to more unique regions used by very few labs. However, due to challenges, such as sequences that are commonly shared by many labs, about 36% of the time an unknown plasmid cannot be successfully narrowed down to only the single correct depositing lab by PlasmidHawk MAX mode. To help ameliorate this difficulty, we have introduced the lab score for inferring the single correct lab-of-origin. The lab score helps order the set of predicted labs from PlasmidHawk MAX mode based on the pan-genome annotation. The incorporation of lab score increases the prediction accuracy from MAX mode’s 64% to CORRECT mode’s 76%.

Of note, PlasmidHawk successfully predicted the correct lab for pCI-YFP as being in the set of labs containing the highest number of aligned fragments using PlasmidHawk MAX mode and further narrows the detection down to the Voigt lab as the true source lab using PlasmidHawk CORRECT mode. In this case, PlasmidHawk has higher accuracy when compared to BLAST, and the same accuracy when compared to the CNN. Furthermore, PlasmidHawk reveals its detailed decision-making process and successfully identifies the signature sequences of the source lab. This human interpretable decision-making process not only yields state of the art performance when analyzing all test set plasmids but also reveals the hidden intricacies of the pCI-YFP classification process. In an actual bioforensics setting, this type of evidence can be both crucial to a correct lab-of-origin assignment, as well as necessary for a proper final justification which is human interpretable. On the other hand, although the deep learning approach can potentially identify the important nucleotides in lab-of-origin detection for specific plasmids using plasmid disruption analysis, it does not intrinsically point out exactly how many labs have used the signature sequences, that is, how unique the subsequences are among all labs.

Our work demonstrates an alignment-based approach can achieve high prediction accuracy while simultaneously providing detailed explanatory capabilities. Meanwhile, we are aware it has potential shortcomings versus a machine learning-based approach. One issue with our method, for instance, is in determining the size and similarity thresholds for creating and adding novel fragments to the synthetic plasmid pan-genome. As plasmid design becomes more and more standardized^29^, sequences from a plasmid can be organized into different modules based on their functions. Researchers can then easily combine different modules to build a variety of plasmids without leaving any editing scars. While this can significantly increase plasmid construction efficiency for synthetic biologists, it can have the unintended consequence of potentially weakening the available signal for determining the lab-of-origin, which may degrade PlasmidHawk’s performance when relying on coarse grained shared fragments as its primary source of signal for predictions. However, if the trend is that synthetic plasmids from distinct labs become more and more similar to each other over time, it likely will become more challenging for PlasmidHawk to rule out labs and accurately predict the true lab-of-origin. Note, this will also be an equally challenging issue for deep learning approaches to handle and affect their ability to detect true depositing labs. On the other hand, as engineered plasmids from given labs become more and more distinct, PlasmidHawk’s performance will increase, specifically related to its accuracy and confidence in its predictions.

Additionally, PlasmidHawk preserves three main advantages over the deep learning approach for lab-of-origin prediction. First, the pan-genome alignment method has the ability to handle large amounts of input synthetic sequences quickly. Although the training time of the CNN and PlasmidHawk are comparable (Supplementary Table 5), PlasmidHawk has potential advantages with respect to updating the model with new plasmids. Whenever a newly engineered sequence is created, the established synthetic plasmid pan-genome can be quickly updated by adding the unique parts of new sequences to the end of the pan-genome and realigning the training plasmids to the new fragments. On the other hand, the CNN model may need to be entirely retrained on the new plasmids if they differ vastly from the previously seen plasmids. Furthermore, it is worth noting that the CNN approach requires additional analysis to determine how well new data fits the existing distribution while the PlasmidHawk approach does not. As more and more synthetic sequences are being added into the database, the computational and environmental cost for retraining neural network approaches will increase alongside^19^. Second, PlasmidHawk is not restricted to predict labs with a small number of deposited plasmids. It has the potential to identify a given plasmid as having originated from a lab that only has one recorded plasmid in the training data set. On the other hand, the CNN requires labs to have enough plasmids in the training set to then be able to be predicted. Third, the pan-genome alignment approach can associate specific regions of an unknown sequence to the source lab. By doing this, PlasmidHawk provides detailed evidence to support its lab-of-origin predictions.

Finally, we have demonstrated an alternative way to characterize research diversity and relationships among synthetic biology labs. By aligning synthetic plasmids from each depositing lab to the synthetic plasmid pan-genome, we are able to capture the resemblances and variations between and within labs engaged in synthetic plasmid engineering. The lab-relatedness tree we have created not only reveals the research structure in synthetic biology, but also implies academic lineage and collaborations. Additionally, it can help researchers to find potential collaborators who work in a similar area. More work on comparative genomics approaches can further assist to trace back the lab-of-origin of an unknown plasmid.

While PlasmidHawk was shown to outperform a previous deep learning method^8^, progress continues to be made in this area. Notably, a recent publication by Alley et al. reported on improved lab-of-origin prediction accuracy up to 70% using a Recurrent Neural Network method called deteRNNt trained on DNA motifs and phenotypes^30^, narrowing the gap of deep learning-based approaches and PlasmidHawk’s performance. While deteRNNt offers substantial gains in accuracy over the previous deep learning approach, PlasmidHawk outperforms deteRNNt with respect to single lab prediction accuracy (76% vs. 70%). For the pCI-YFP plasmid example, PlasmidHawk correctly reports the Voigt lab while deteRNNt returns two labs (the Voigt Lab and Wang Lab, ~25% probability to each). PlasmidHawk’s performance is based on DNA sequence alone compared to both DNA motifs and six phenotypes used by deteRNNt (bacterial resistance, copy number, growth strain, growth temperature, selectable markers, and species).

In conclusion, PlasmidHawk is a computational approach to leverage sequence alignment and annotated pan-genomes for the specific research task of identification of lab-of-origin. Overall, the aim of our study was not to diminish the important achievements of deep learning in lab-of-origin analyses, but rather point out the value of investigating traditional comparative genomics methods such as by using genome alignment. On the whole, this paper intends to encourage the community to keep in mind traditional sequence alignment algorithms coupled with recent advances in pan-genomic analyses, in spite of recent exciting advances in AI/ML applied to computational biology. We anticipate future advances will combine the benefits of both well studied and interpretable methods like genome alignment with the power of deep learning models such as the CNN model of Nielsen et al. or RNN model of Alley et al. for improved biosecurity of engineered DNA.

## Methods

### Addgene dataset

We acquired a plasmid dataset from Addgene in January 2019. Addgene is a synthetic plasmid repository. It was used in Nielsen et al. to conduct the deep learning lab-of-origin prediction study. DNA sequences in Addgene can be classified into four categories: full plasmid sequences submitted by Addgene, partial sequences submitted by Addgene, full plasmid sequences submitted by a depositing lab, and partial sequences submitted by a depositing lab. There are a total 51,047 complete sequences, in which 28,879 are uploaded by Addgene, and 737,272 partial sequences. The DNA sequences and their metadata are stored in a JavaScript Object Notation (JSON) file. In Nielsen et al., a plasmid depositing lab was parsed directly from the JSON file. However, the JSON file we obtained had no deposting lab information. To decide a plasmid’s depositing lab, we first found information from its Genbank file. We took the last author in the first published paper of the plasmid as the depositing lab. For the plasmids without Genbank files, we looked up the author information through its PubMed ID (PMID) or PMCID in the JSON file. If we still could not find its depositing lab, we parsed the depositor information directly from the Addgene website.

### PlasmidHawk workflow

The main goal of our study is to predict engineered plasmids’ lab-of-origin by aligning unknown plasmids to a synthetic plasmid pan-genome. In order to do that, we developed a lab-of-origin prediction software called PlasmidHawk. It consists of three modules: pan-genome construction, pan-genome annotation, and lab-of-origin prediction. In general, the three modules should be used sequentially for plasmid lab-of-origin detection. Each module can also be applied independently for other scientific purposes.

For the first module, PlasmidHawk takes in plasmids from the Addgene database and constructs a synthetic plasmid pan-genome *P* using Plaster. Plaster is a state-of-the-art linear pan-genome construction algorithm^26^. The final pan-genome *P* is composed of a set of sequence fragments *F* = [*f*_0_, *f*_1_, . . . *f*_*n*_]. Each fragment is at least 50 bp long (default) and stored as an independent entry in a multi-fasta file. After building the synthetic plasmid pan-genome, PlasmidHawk aligns input plasmids back to the pan-genome. If a pan-genome fragment has at least 20 bp (Nucmer default parameters) matching with sequences from an input plasmid, the fragment is annotated with that plasmid’s depositing lab.

**Fig. 4:**
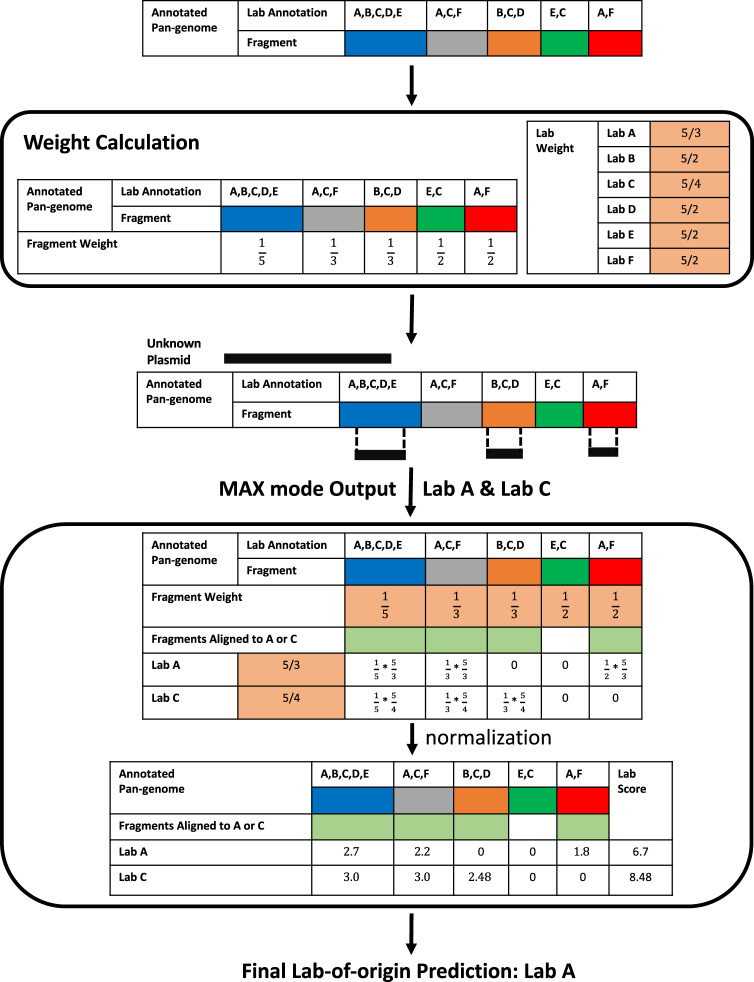
PlasmidHawk CORRECT mode workflow. After building the reference pan-genome, CORRECT mode calculates fragment weights and lab weights according to the pan-genome annotations. To predict the lab-of-origin of an unknown sequence, PlasmidHawk first aligns the query sequence to the pan-genome and identifies candidate source labs through MAX mode: in this case, lab A and lab C. CORRECT mode then calculates lab scores for the labs output from MAX mode. In the end, CORRECT mode predicts the lab with the minimum lab score, lab A, as the depositing lab.

To predict the lab-of-origin of an unknown plasmid *p*, PlasmidHawk aligns the plasmid *p* to the reference pan-genome *P* built in the first step. It then extracts aligned pan-genome fragments from the pan-genome. Each aligned pan-genome fragment has a match of at least 20 bp with the plasmid *p*. PlasmidHawk MAX mode then outputs lab(s) with the highest number of aligned fragments as the predicted source lab(s). To further improve the lab-of-origin prediction accuracy, PlasmidHawk CORRECT mode calculates lab scores for labs returned from PlasmidHawk MAX mode. CORRECT mode takes lab(s) with the minimum lab score as the final prediction as explained in full detail in the following section and visualized in Fig. 4. Briefly, the value of a lab’s score depends on the total number of pan-genome fragments each lab has and the number of labs sharing the same aligned fragments. Mathematically, the lab score for lab *l*, denoted as *S*_*l*_, is

1 $${S}_{l}=-\sum _{f\in {F}_{l}}{\mathrm{{log}}}\left(\frac{1}{{n}_{f}* {t}_{l}}\right)$$

where *F*_*l*_ is the aligned fragments set for lab *l*. It includes all the aligned fragments lab *l* has for the query sequence. *n*_*f*_ is the number of labs sharing fragment *f* in the pan-genome *P*. And *t*_*l*_ is the total number of fragments lab *l* has in *P*.

### MAX mode *p* value

The fundamental mechanism of PlasmidHawk MAX mode is to identify labs that are enriched by the pan-genome fragments that align with the query sequence. This question is similar to gene set enrichment analysis, which focuses on finding a subset of genes that are over-represented in a large set of genes, and may be correlated with certain phenotypes. In gene enrichment analysis, the hypergeometric *p*-value is widely used in quantifying whether enriched genes with pre-defined functional annotations are significant or not. Inspired by this approach, and given how well the hypergeometric distribution matches our own problem, we apply the hypergeometric *p*-value to evaluate the significance of predicted labs by PlasmidHawk MAX mode. However, the hypergeometric *p*-value used in this study has several limitations. A detailed discussion is included in Supplementary Note 4.

In our case, we model the number of fragments in the pan-genome as the finite population size *N* for the hypergeometric distribution. From this population, we perform *n* draws, where *n* is the number of fragments in a query plasmid. We then define, based on the most frequent lab as predicted by MAX mode, the number of successes *k* as the number of fragments in the query plasmid that aligned to any of the *K* fragments in the pan-genome that were annotated as having come from that most frequent lab. The final *p*-value is then calculated using the python package scipy.stats.hypergeom^31^, calculating the *p*-value of the hypergeometric distribution when observing *k* or more successes.

### Lab score

After obtaining a list of potential source labs from MAX mode, PlasmidHawk CORRECT mode attempts to further narrow down the labs to the true source lab. To do that, it calculates lab scores to rank labs returned from MAX mode as shown in Fig. 4. Essentially, lab scores are assigned to individual labs through a weighting function. Labs with lower lab scores have a higher chance to be the real depositing labs. The weighting function used to calculate lab scores is derived from our key observations that despite the maximum number of aligned fragments being the same among multiple labs, pan-genome fragments shared by many labs are potentially less informative versus fragments shared by few labs. Also, labs with less total fragments in the pan-genome can be weighted higher when making the final predictions.

Specifically, after constructing and annotating the reference pan-genome, CORRECT mode first calculates weights for each lab, denoted as *W*_*l*_ for lab *l*, and each fragment, referred as *W*_*f*_ for fragment *f*, based on the pan-genome annotations. The lab weight

2 $${W}_{l}=\frac{T}{{t}_{l}}$$

where *T* is the total number of fragments in the reference pan-genome and *t*_*l*_ is the total number of fragments lab *l* has in the pan-genome. *W*_*l*_ is the reciprocal of the fraction of fragments annotated by lab *l* inside the pan-genome. The fragment weight

3 $${W}_{f}=\frac{1}{{n}_{f}}$$

where *n*_*f*_ is the total number of labs annotated to fragment *f* (Fig. 4).

To calculate the final scores used for prediction, CORRECT mode goes through all the labs returned by MAX mode and all the aligned fragments for each of these labs and calculates a joint weight *W*_*l*,*f*_ for each aligned fragment *f* and lab *l*. To calculate *W*_*l*,*f*_, PlasmidHawk first identifies the set of aligned fragments *F*_*l*_ for each lab *l* identified by MAX mode. CORRECT mode then calculates *W*_*l*,*f*_ by multiplying *W*_*l*_ and *W*_*f*_, and normalizing it with its maximum possible value (*T*). The normalization bounds *W*_*l*,*f*_ between 0 and 1. Mathematically,

4 $${W}_{l,f}={W}_{f}* {W}_{l}/T=\frac{1}{{n}_{f}* {t}_{l}}$$

CORRECT mode then does a log transformation of each joint weight to avoid multiplication operations and potential overflows when calculating the final single fragment lab score *S*_*l*,*f*_. It adds a final negative sign to each transformed value to make the final scores positive.

5 $${S}_{l,f}=-{\mathrm{{log}}}({W}_{l,f})$$

Finally, CORRECT mode sums the *S*_*l*,*f*_ of all the fragments in *F*_*l*_ to generate the final lab score *S*_*l*_ for lab *l* used for the final prediction. The lab with the lowest *S*_*l*_ is chosen as the predicted lab-of-origin as outlined in Fig. 4.

6 $${S}_{l}=\sum _{f\in {F}_{l}}({S}_{l,f})$$

### PlasmidHawk lab of origin prediction

Given the nature of neural networks, the CNN needs a certain number of plasmids from each lab to train, test and validate the neural network. Although PlasmidHawk does not have this kind of requirement, in order to have a fair comparison between the CNN and PlasmidHawk, we only choose labs with at least 10 complete plasmids (“Full Repository” or “Full Depositor”) sequences in Addgene to conduct this experiment. A total of 38,681 plasmid sequences from 896 labs are used in this experiment. To evaluate the performance of PlasmidHawk, sequences are split into two groups: three plasmids from each lab are randomly selected for testing and the remaining plasmids are used for the training set. We utilize the training plasmids to build and annotate the synthetic plasmid pan-genome. We evaluate PlasmidHawk prediction accuracy using plasmids in the test data set. The entire process is repeated five times.

To assess the lab-of-origin prediction accuracies of MAX mode and CORRECT mode, we test PlasmidHawk at different thresholds; we test the accuracy when considering the top 1, 2, 5, 10, 20, and 50 labs output from PlasmidHawk. For MAX mode, we only consider the top predictions at or above the threshold, and eliminate sets of predictions whose inclusion would cause there to be more predictions than the threshold. For CORRECT mode, we only consider the scored, ordered list created from the top set of predictions from MAX mode. For example, when setting the threshold at 1, for MAX mode, we only consider correct predictions when the top set of predicted labs contains only a single lab and that is the correct depositing lab. If MAX mode outputs more labs in the top set of predictions than the threshold, no labs are considered and the prediction is considered incorrect, even if the correct lab is in the top set of predictions. For CORRECT mode with a threshold of 1, we order the top set of MAX mode predictions, and only consider the single best scoring prediction. As another example, when setting the threshold at 5, and MAX mode outputs a set of two labs as the top predictions, two labs in the second best set of predictions, and two labs in the third best set of predictions, the four labs in the top two sets would be considered and the two labs in the third set would not be considered. In this and all other cases, CORRECT mode considers only the top set of labs from MAX mode, thus for the top 5 threshold it would still only consider the ranked list of the top two labs from MAX mode. In addition, if a set of labs in CORRECT mode have the same lab score, we arbitrarily order them and apply the threshold, then add them into our final prediction lists. By doing this, the number of labs in a CORRECT mode prediction result equals the threshold. For instance, if the threshold is 1 and there are two labs with the same lab score returned from CORRECT mode, we will arbitrarily select one lab as the CORRECT mode prediction.

While building the pan-genome, we use Plaster, which can be installed via “conda install pan-plaster”. For PlasmidHawk, we use default setting. The example command lines are available in gitlab repository.

### CNN architecture and lab of origin prediction

The CNN architecture was constructed based on Nielsen et al. All the CNN parameters are set to their optimized values from Nielsen et al.. In the original experimental design, the authors reported splitting the data set into six subsets because of memory limitations. We replicate this by separating the training data into six subsets, then load and train one at a time in each epoch. After training, we save the final model and parameters.

We use the same plasmid data set from the PlasmidHawk experiments to train, validate, and test the CNN approach. We randomly pick three plasmids from each lab as the validation data set and then pick an additional three plasmids as the test data set. The remaining plasmids are used as the training set. We preprocess and encode the DNA sequences. As in Nielsen et al., we set all the DNA sequences to 8000 bp long by truncating the long DNA sequences and padding the short DNA sequences with Ns. Characters other than A, T, C, or G in the DNA sequences are converted to Ns. We append the sequence’s reverse complement to itself with 48 Ns in between. We translate those processed sequences as a one-hot vector with length 16,048 where A = [1000], T = [0100], G = [0010], C = [0000]. The depositing lab is encoded as a one-hot vector with total length 896. This experiment is repeated five times.

To evaluate the CNN prediction accuracy, we calculate the percentage of plasmids in the test data set correctly identified their lab-of-origin while considering the top 1, 2, 5, 10, 20, and 50 predicted labs from CNN. We then compute the average percentages of correct predictions and their standard deviations at different thresholds (1, 2, 5, 10, 20, 50).

### Receiver operating characteristic (ROC) curve for CNN and PlasmidHawk

To calculate the ROC curve for the CNN and PlasmidHawk, we first need to define what constitutes a true negative (TN), true positive (TP), false negative (FN), and false positive (FP). We then calculate the average true positive rate and false positive rate at different thresholds (top *N* predicted labs). For a given testing plasmid, if a lab is within the top *N* cutoff, the lab is a TP if the lab is the true depositing lab and a FP otherwise. If a lab is not within the top *N* cutoff, then it is a FN if the lab is true depositing lab and a TN otherwise.

### Averaged lab Jaccard distance and averaged lab score

**Fig. 5:**
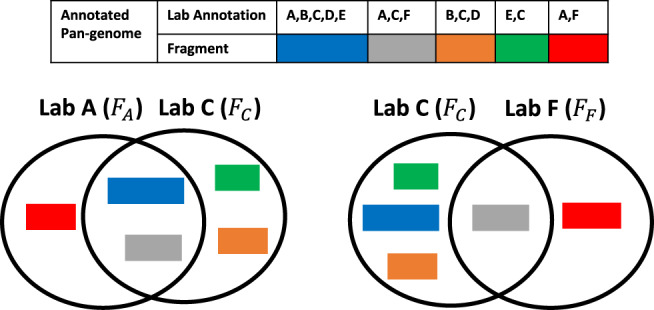
Lab Jaccard distance calculation. To calculate lab Jaccard distances between two labs, such as lab A and lab C, we first build a fragment set, *F*_*A*_ and *F*_*C*_, for each lab. A fragment set contains all the pan-genome fragments annotated by the corresponding labs. The lab Jaccard distance between lab *A* and lab *C* is *JD*(*A*, *C*) = 1 − *J*(*F*_*A*_, *F*_*C*_), where the Jaccard index (*J*(*F*_*A*_, *F*_*C*_)) is the fraction of shared pan-genome fragments out of all the different fragments lab A and C have. A large lab Jaccard distance indicates two labs have few shared sequences.

To evaluate the relationships between lab scores and the uniqueness of labs’ plasmids, we calculate the lab Jaccard distance between all labs. The lab Jaccard distance quantifies the sequence similarities between all plasmids between two labs. To measure lab Jaccard distances, we first build and annotate a complete synthetic plasmid pan-genome using all plasmids from labs who have at least 10 complete sequences. We then extract all the fragments annotated with lab *A* to fragment set *F*_*A*_. We do this again for all labs. We define the lab Jaccard distance between two labs, lab *A* and lab *B*, as

7 $${\mathrm{{JD}}}(A,B)=1-J({F}_{A},{F}_{B})$$

where

8 $$J({F}_{A},{F}_{B})=\frac{| {F}_{A}\cap {F}_{B}| }{| {F}_{A}\cup {F}_{B}| }$$

represents the Jaccard index between two labs (Fig. 5). We built a distance matrix between all labs by calculating the pairwise Jaccard distances between every pair of labs. This distance matrix was used, for instance, to build the lab-relatedness tree and also to calculate the “averaged lab Jaccard distance” for each individual lab. The averaged lab Jaccard distance for a lab is simply the average of all the cells in the corresponding row or column for that lab in the distance matrix.

To calculate a lab’s “averaged lab score”, we first run CORRECT mode on all test plasmids from the five independent lab-of-origin prediction experiments. If CORRECT mode returns a score for the true depositing lab for a plasmid, we assign that returned lab score to that plasmid. A lab’s “averaged lab score” is the average of all assigned lab scores for all test plasmids corresponding to that lab.

### pCI-YFP prediction analysis

To identify pCI-YFP’s depositing lab, we input the pCI-YFP sequence, the complete synthetic pan-genome sequence, and the pan-genome annotation information into PlasmidHawk. PlasmidHawk returns the predicted labs and their lab scores. It also outputs alignment results. We retrieve the aligned pan-genome fragments of pCI-YFP and the list of labs having those aligned fragments to create the alignment plot (Fig. 3b).

### Lab-relatedness tree

We apply RapidNJ^32^ to construct a lab-relatedness tree with support values based on lab Jaccard distances. RapidNJ employs the neighbor-joining method^33^ to build trees. In order to compute bootstrapping support value for the lab tree, we build a binary matrix for the plasmid fragments used by each lab. The columns of the binary matrix represent all fragments in the pan-genome. The rows of the matrix represent each of the labs. Each row in the matrix can be viewed as a binary vector for a particular lab, where each entry indicates the presence or absence of a particular pan-genome fragment within that lab’s plasmids. If a lab *i* has a fragment *j* in the pan-genome, the cell in the *i*th row and *j*th column is a 1 in the matrix. Otherwise, the cell is 0. To make a bootstrap replicate of the original matrix, a new matrix of the same size is created by randomly sampling the columns from the original matrix (with replacement). For this study, 100 bootstrap replicate matrices are generated. After that, we calculate pairwise Jaccard distances between any two labs for each bootstrap replicate, and build the corresponding lab-relatedness tree. A final comparison of the topologies of the 100 replicate trees to the original tree is done to add support values. This procedure is performed using the python Bio.Phylo package^34^.

The visualization is conducted with the interactive tree of life (https://itol.embl.de)^35^. The full lab-relatedness tree can be viewed in: http://itol.embl.de/shared/qiwangrice.

### Statistical analysis

The principal component analysis is conducted using the sklearn.decomposition function^36^. The explained variances for PC1 and PC2 are 1.48 and 0.92. Linear regression is performed using the *sklearn*. *linear*_*model* function^36^. All the code is available in the GitLab repository^37^.

### Reporting summary

Further information on research design is available in the Nature Research Reporting Summary linked to this article.

## Supplementary information

Supplementary Information

Reporting Summary

## Data Availability

The commands used and the source code are available on GitLab. Due to data sharing constraints, we are not permitted to redistribute the plasmid DNA sequences deposited in AddGene’s repository. The plasmid sequences are available individually from the AddGene website (https://www.addgene.org/browse/) for download, and available for bulk download from AddGene upon request. Intermediate data is available by request and all of the methods, experimental results and scripts are open source and available on GitLab (https://gitlab.com/treangenlab/plasmidhawk.git). The DOI of the repository is 10.5281/zenodo.4405001. The pCI-YFP plasmid is available from Genbank via Accession JQ394803.1.

## Code availability

PlasmidHawk is written in Python and is available at https://gitlab.com/treangenlab/plasmidhawk.git. All the scripts, including *p*-value calculation, used in this study to generate results and figures are available at https://gitlab.com/treangenlab/plasmidhawk/-/tree/data^37^. The DOI of the repository is 10.5281/zenodo.4405001.
