# Supplementary material for: PlasmidHawk improves lab of origin prediction of engineered plasmids using sequence alignment
Source: Nat Commun. 2021 Feb 26;12:1167. doi: 10.1038/s41467-021-21180-w (PMC7910462; doi:10.1038/s41467-021-21180-w)
Supplement: Supplementary file 1 — Supplementary Information [file 41467_2021_21180_MOESM1_ESM.pdf]

Supplementary Material

Supplementary Note 1 To demonstrate the usefulness of PlasmidHawk in the case where a query plasmid's true depositing lab is completely missing from the plasmid training data, we ran PlasmidHawk to predict two randomly selected plasmids: plasmids 116938 from the Holt J lab and 105687 from the Hegde RS lab. Because both labs have less than 10 plasmids in Addgene, none of their plasmids were used in building our pan-genome, that is, their plasmids were not in the training plasmids.

For plasmid 116938, PlasmidHawk MAX mode mode predicts the Jennifer Doudna lab as the true depositing lab. Plasmid 116938 has 54 pan-genome fragments and 44 out of 54 pan-genome fragments are shared with Jennifer Doudna. Among all the pan-genome fragments shared by both plasmid 116938 and the Jennifer Doudna lab, one fragment was annotated by only 12 labs, which align to a specific ribosome binding region (RBS). Additionally, we found that the origin of replication and the insertion region just before the gene bom also play a role as they both have fragments with less than 100 annotated labs.

In the next example, PlasmidHawk predicts the Michael Davidson lab as the plasmid 105687 lab-of-origin. The query plasmid maps to 93 pan-genome fragments and 46 of them annotated with Michael Davidson. Based on PlasmidHawk's alignment results, CMV enhancer (109-227nt), EGFP(561-1267nt), and mCherry(1886-2593nt) are three important regions in identifying the lab-of-origin for plasmid for 105687. The pan-genome fragments, which align to the aforementioned regions, are each found in less than 100 labs, while the Michael Davidson lab has all of these fragments.

Supplementary Note 2 Previously, we applied the averaged lab Jaccard distances to quantify the uniqueness of a lab's sequences. These data can be further explored to measure the research diversity within and between labs. Here, we introduce two kinds of diversity measurements: between-lab research diversity score and within-lab research diversity score. Between-lab research diversity score describes the dissimilarities of plasmids from a given lab compared to other labs. It can be quantified by the averaged lab Jaccard distances. The larger the distance is, the more distinct a lab's sequences are from other labs' sequences.

The within-lab research diversity score (WRDS) refers to the labs own research diversity - the similarity among sequences within their own labs. WRDS can be quantified by the number of fragments each lab has in the pan-genome. A lab using more pan-genome fragments tends to have more variety among its plasmids. Based on this definition, the Hahn Klaus lab has the largest within-lab research diversity score (Supplementary Table 1).

The obvious problem with the definition of WRDS is that labs with a large number of plasmids are more likely to have a large number of fragments (higher WRDS). Therefore, we normalize the WRDS with the number of plasmids each lab has. This is referred to as the normalized within-lab research diversity score (nWRDS). Labs with larger nWRDS values, on average, use more unique subsequences to construct their plasmids. The Zhang YZ lab has the largest nWRDS value. ZHang YZ has a total of 11 plasmids, which align to 129 different fragments. On average, each plasmid can align to 11.7 different fragments (Supplementary Table 2). On the other hand, David Root has a total of 2717 plasmids and all those plasmids are aligned to 108 fragments in the complete pan-genome. Therefore, the David Root lab has the smallest nWRDS value of only 0.04 (Supplementary Table 3). As we mentioned previously, most of the Root David plasmids are from single library screening papers. This lowers the total number of fragments each plasmid aligns to, since most plasmids will align to the same set of fragments in the complete pan-genome. In the future, we could collapse plasmids from the same library into a single plasmid to calculate nWRDS value to correct for this.

Supplementary Table 1. Top 10 labs with the highest within-lab research diversity score (WRDS) .

Lab	Within-lab Research Diversity (Number of Fragments)
Hahn Klaus	771
Weiss Ron	716
Lu TK	444
Church George	414
Voigt CA	385
Zhang Feng	382
Luo LLiqun	370
Sidhu	352
White Kevin	352

Supplementary Table 2. Top 5 labs with the highest normalized within-lab research diversity score (nWRDS).

Lab	Normalized Within-lab Research Diversity
Zhang YZ	11.7
Cook JG	11.2
Varshavsky Alexander	9.7
Weiner Orion	9.3
Alvarado-Kristensson M	9.2

Supplementary Table 3. Bottom 5 labs with the lowest normalized within-lab research diversity score (nWRDS).

Lab	Normalized Within-lab Research Diversity
Root David	0.04
Barrett LE	0.09
Cheeseman Iain	0.22
Bennett EP	0.32
Ekker SC	0.35

Supplementary Table 4. Collaboration between labs in the clade.

Collaborator	Evidence of collaboration
Voigt CA; Lu TK	https://doi.org/10.1016/j.cels.2015.06.001
Liu DR; Church George	US20080051317A1
Liu DR; Zhang Feng	https://pairwise.com/about-us/founders/
Lu TK; Church George	PMC2690711

Supplementary Table 5. Training time of the CNN and PlasmidHawk

Time (min)	CNN	PlasmidHawk
User	19,262	21,048
System	2,223	253.9
Total	21,485	21,302

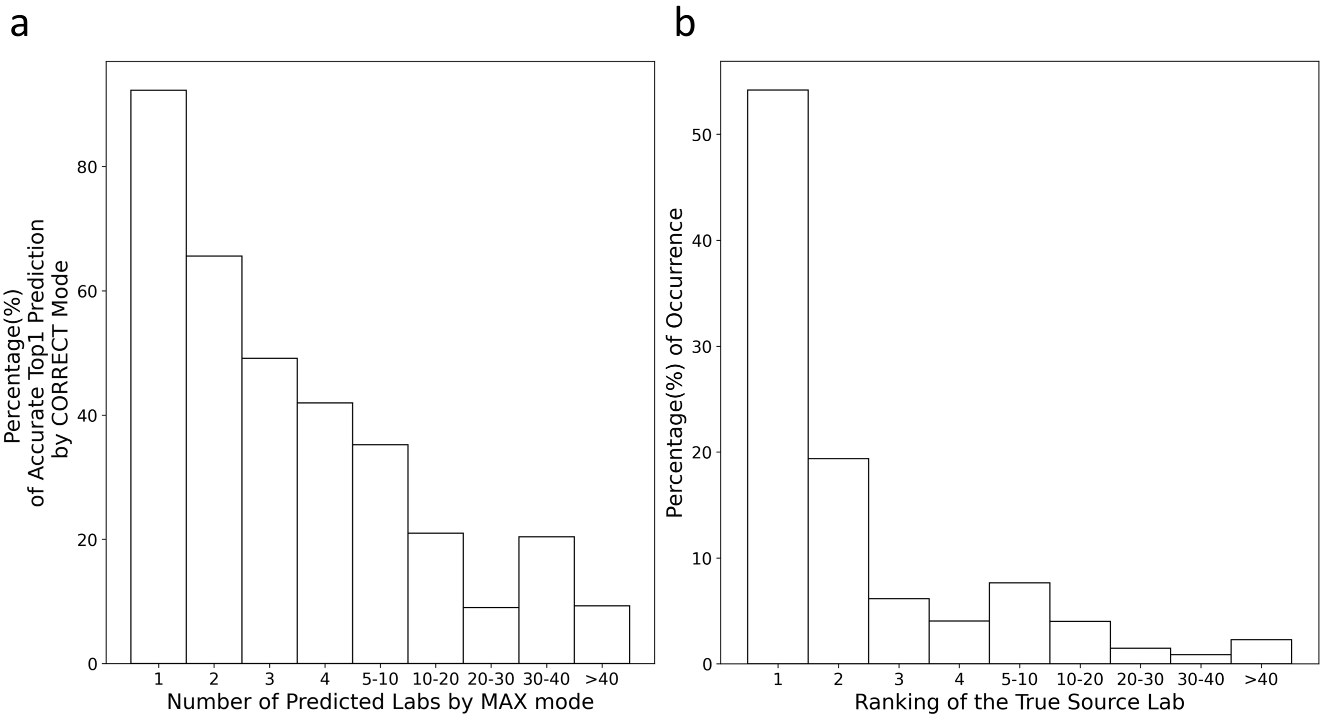

Supplementary Figure 1. **a** Percentage of times that CORRECT mode predicted the true lab-of-origin versus the number of top labs identified by PlasmidHawk MAX mode. The X-axis represents the number of labs predicted by MAX mode. The y-axis represents the percentage of times CORRECT mode's top 1 prediction was correct in that interval. **b** The distribution of true source lab rankings when it is within the labs identified and ranked by CORRECT mode. The X-axis represents the ranking of the true depositing lab based on the lab scores output by CORRECT mode. The y-axis represents the percentage of times that this occurs out of all cases where the correct source lab is included in the output of CORRECT mode.

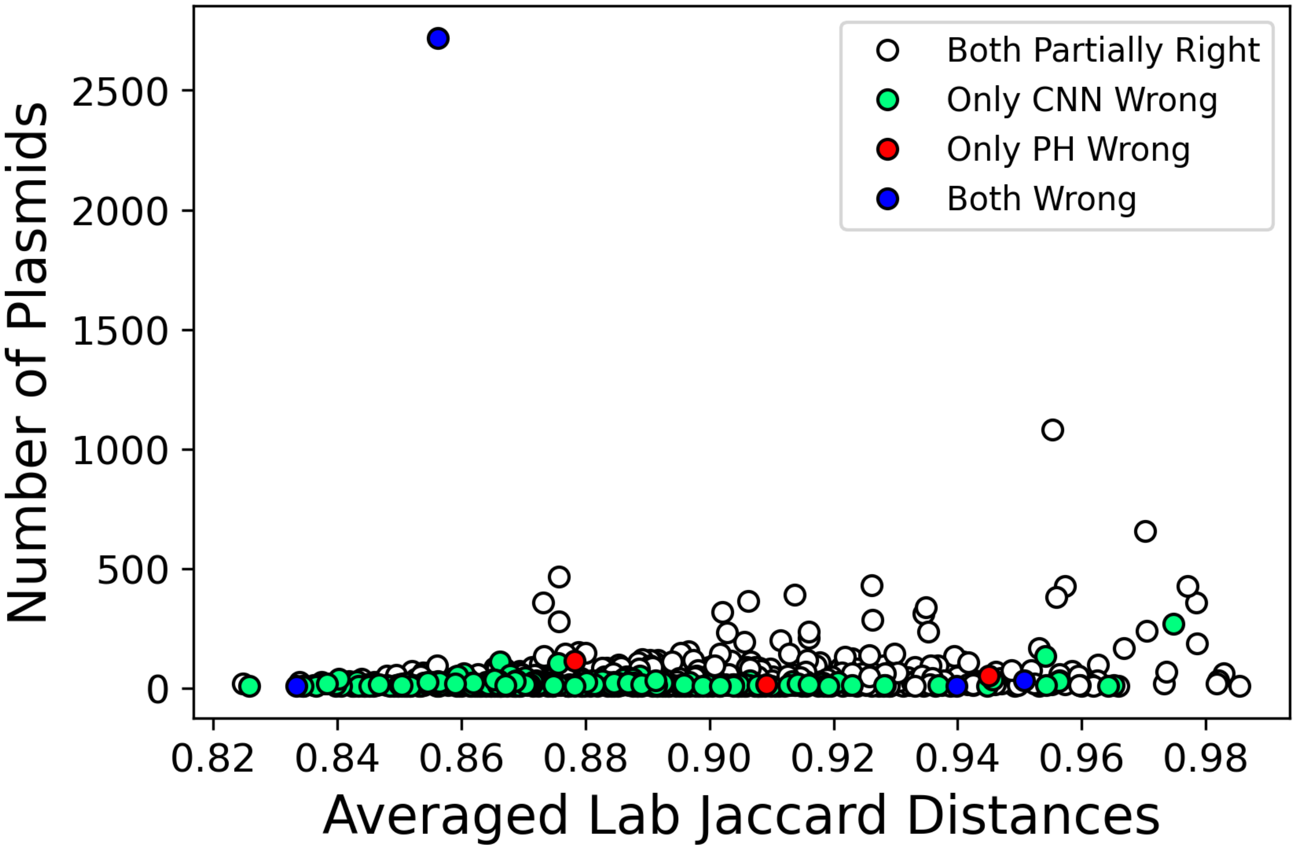

Supplementary Figure 2. Averaged lab Jaccard distances and number of plasmids each lab has. Each dot represents a lab. The x-axis is the averaged lab Jaccard Distance and the y-axis is the number of plasmids each lab has. Blue dots represent labs where all of their plasmids in five experiments are predicted wrong by both the CNN and PlasmidHawk. Red dots represent labs where all of their plasmids are predicted wrong only by PlasmidHawk. Green dots represent labs whose testing plasmids are predicted wrong only by the CNN. White dots represent labs with at least one testing plasmid correctly predicted by the CNN and PlasmidHawk.

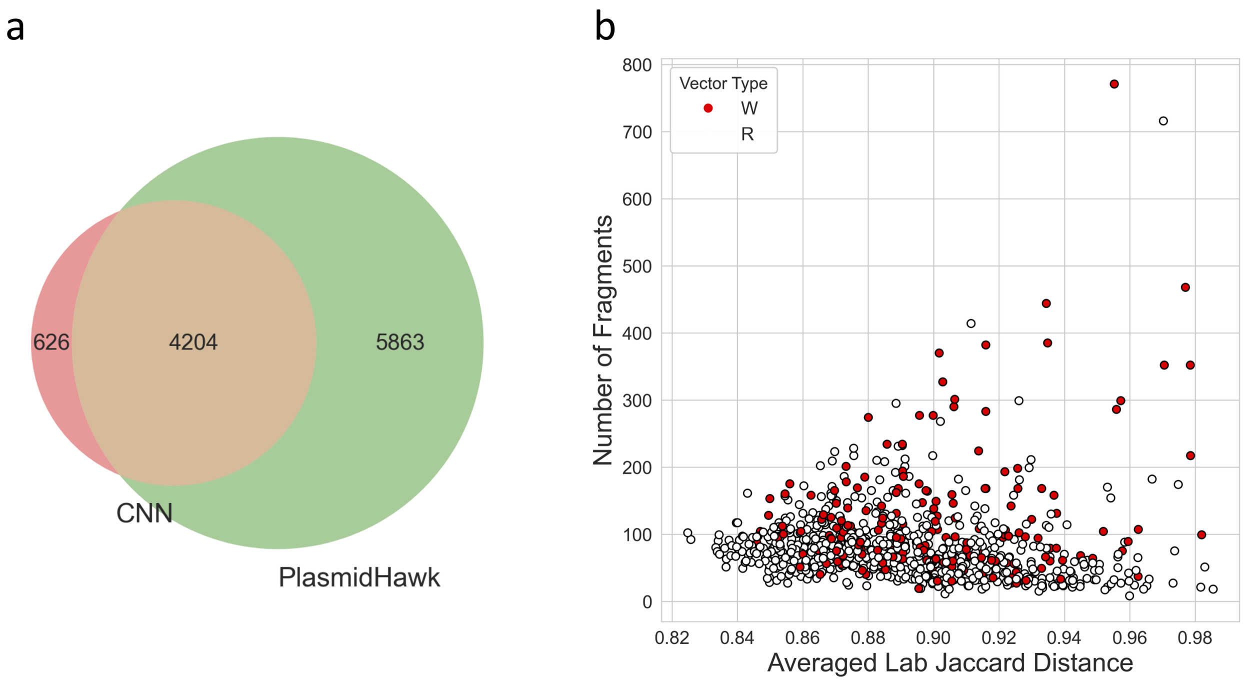

Supplementary Figure 3. **a** The number of testing plasmids predicted correctly by the CNN and PlasmidHawk (top 1 prediction). The red ball represents the number of plasmids predicted correctly only by the CNN and the green ball represents the number of plasmids predicted correctly only by PlasmidHawk. The yellow region represents the number of plasmids whose lab-of-origin are successfully identified by both the CNN and PlasmidHawk. **b** Averaged lab Jaccard distances and number of fragments each lab has. Each dot represents a lab. The red dots represent labs with at least one plasmid that is correctly predicted by the CNN approach, but not by PlasmidHawk.

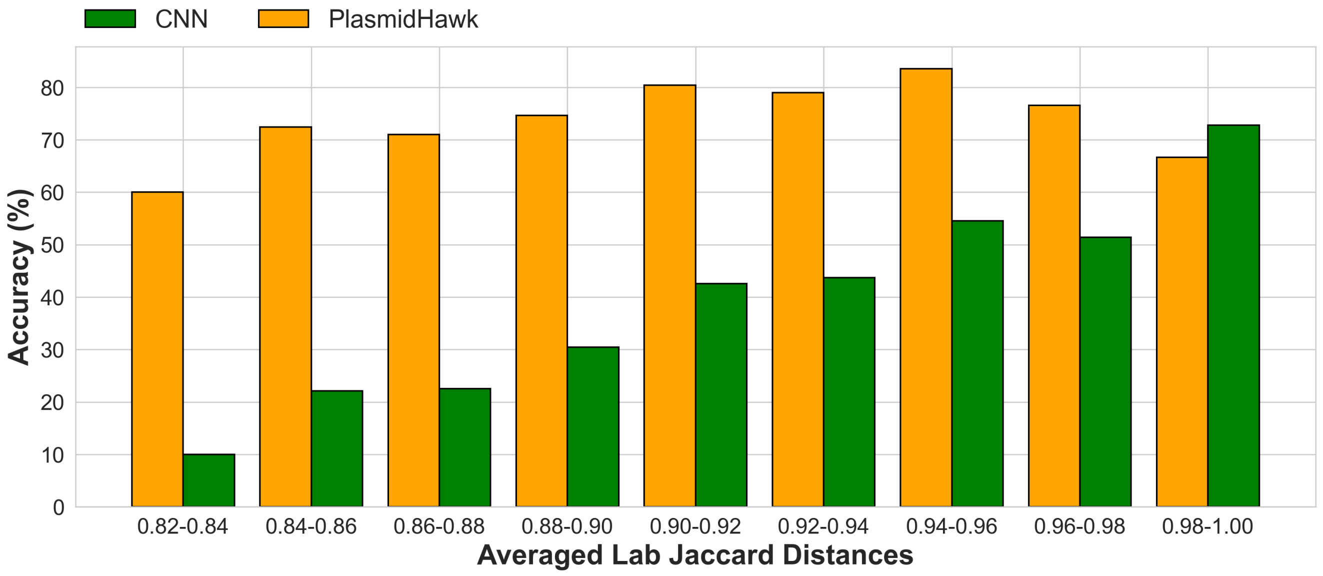

Supplementary Figure 4. Lab-of-origin prediction accuracy for labs with different averaged lab Jaccard distances. The X-axis shows labs with different averaged lab Jaccard distances and the y-axis is the prediction accuracy of the testing plasmids from those labs.

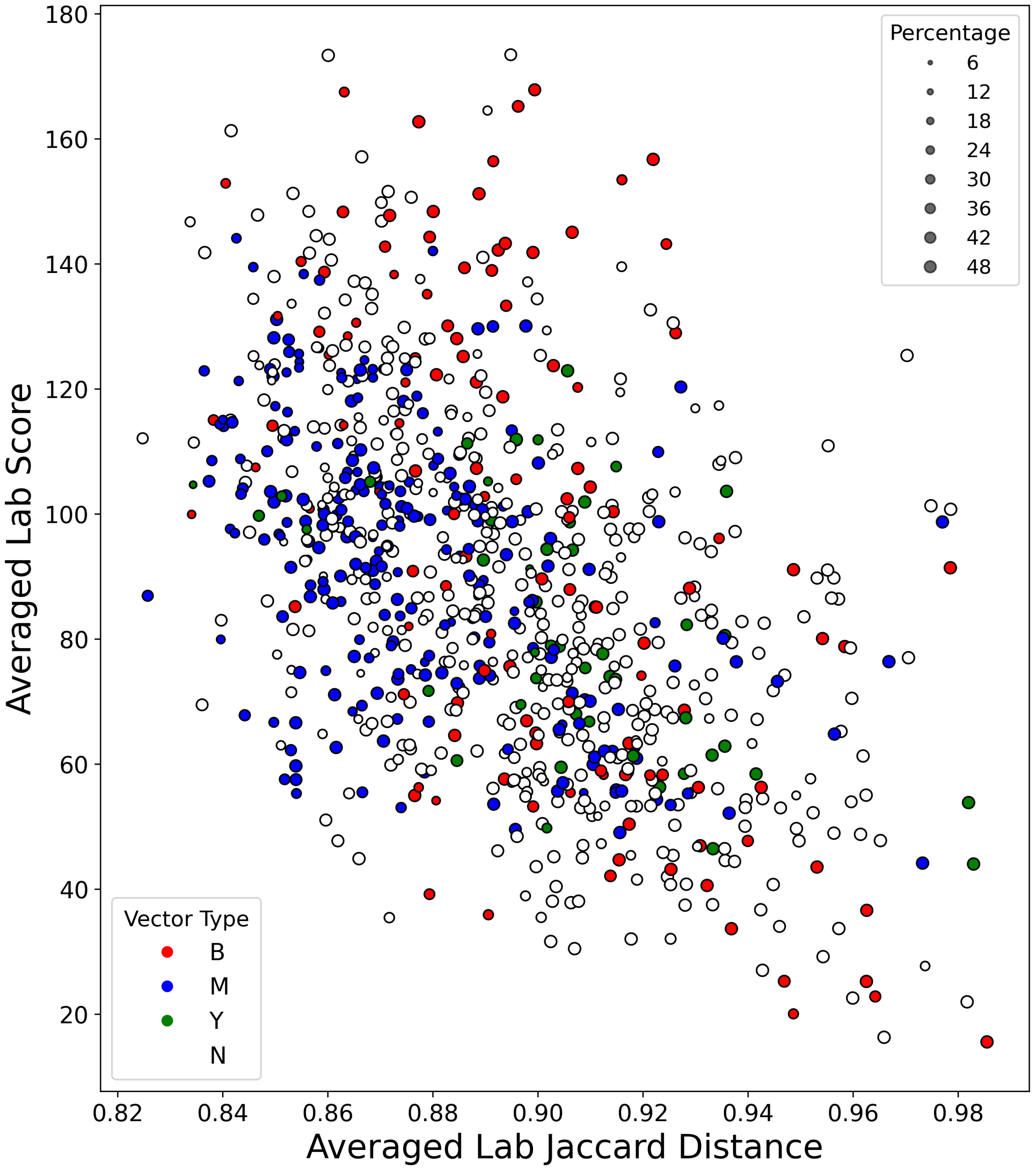

Supplementary Figure 5. The distribution of different lab types visualized over the averaged lab Jaccard distance and averaged lab score for each lab.The colors label labs based on their sequences' host cells. The size of the dot corresponds to the percentage of the most abundant host cells inside a lab. Blue: mammalian lab (M), Green: yeast lab (Y), Red: bacterial lab (B), black: N/A lab (NA).

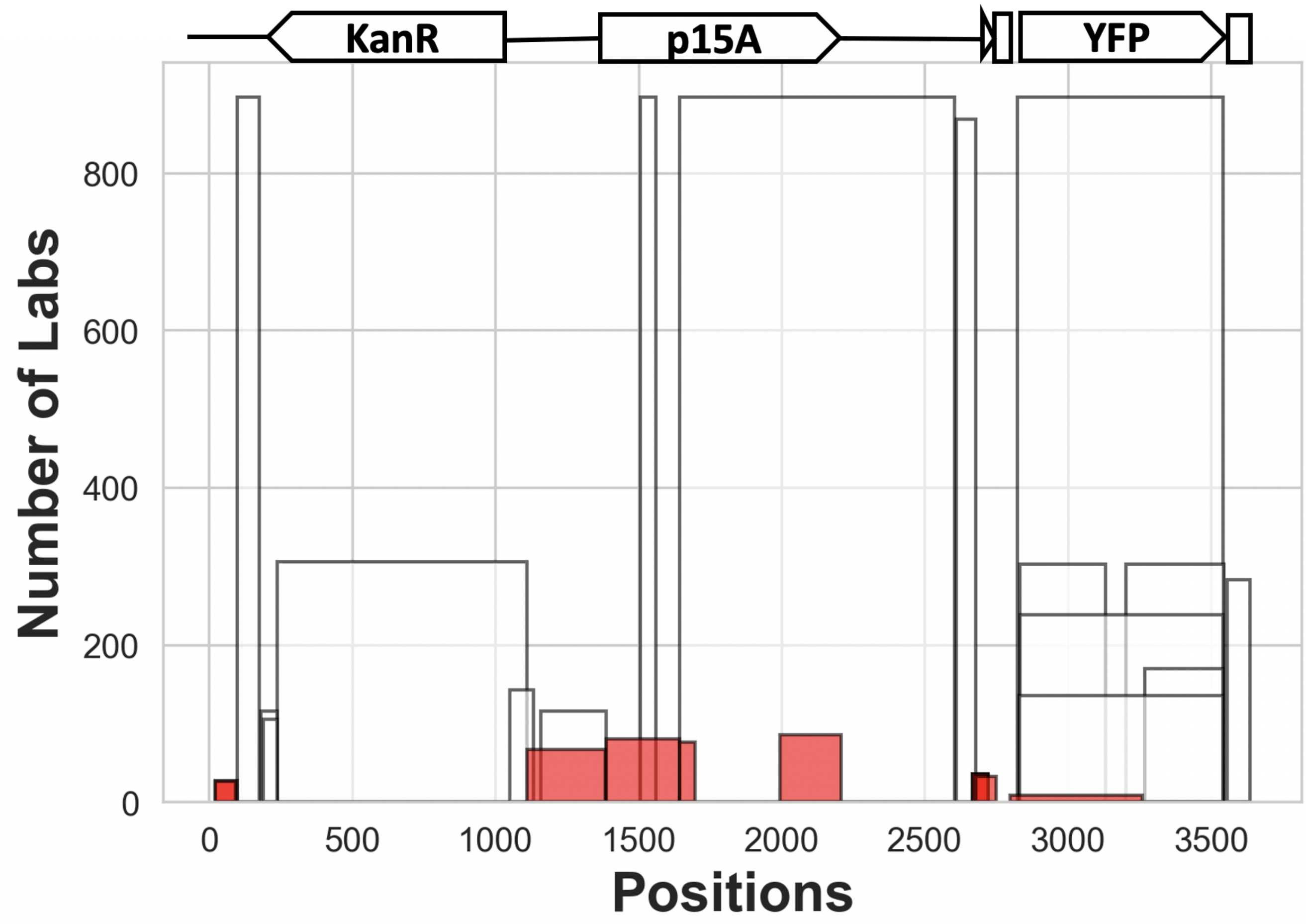

Supplementary Figure 6. The number of labs annotated for the regions of pCI-YFP that aligned to different fragments in the synthetic pan-genome. Each bar represents a fragment aligning to the positions in pCI-YFP. The height of the bar represents the number of labs annotated with that fragment. Pan-genome fragments with less than 100 total lab annotations have aligned to pCI-YFP at 21-98nt, 1110-1697nt, 1993-2209nt, 2667-2751nt and 2797-3260nt positions (red bars).

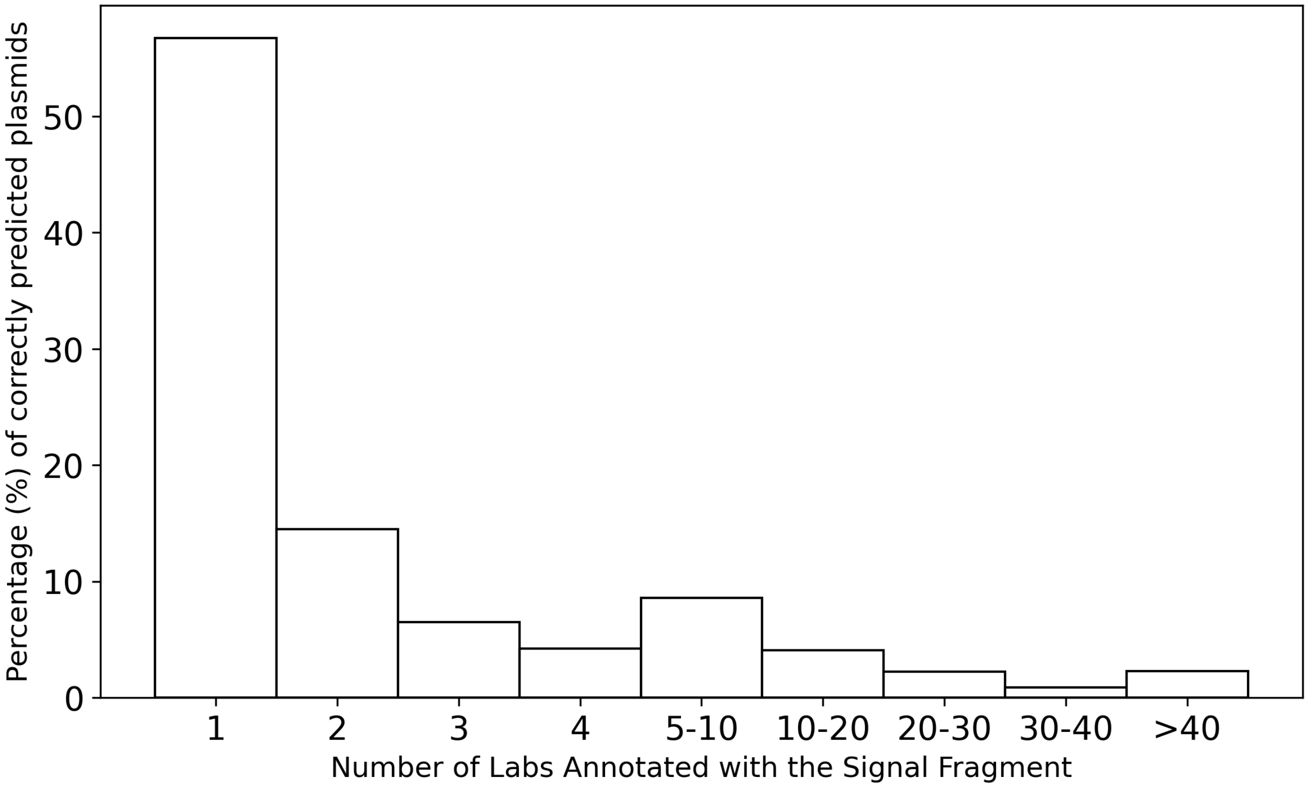

Supplementary Figure 7. Percentages of correctly identified plasmids with different types of signal fragments. The X-axis shows different types of signal fragments binned based on the number of labs annotated to them. The Y-axis shows the percentage of successfully predicted plasmids with the different types of signal fragments.

Supplementary Note 3 To understand the kind of techniques that are easily traceable, we need to identify the signature sequences that help PlasmidHawk identify the depositing labs. To do that, we looked at all the testing plasmids whose lab-of-origin are successfully identified by PlasmidHawk CORRECT mode mode with a single shot, and the pan-genome fragments those plasmids aligned to. Based on the algorithm of PlasmidHawk, pan-genome fragments with a smaller number of labs annotated are considered as signature sequences with important lab signals. Thus, we examine the pan-genome fragments, which overlap with the query sequences, and refer to fragments with the least number of labs annotated to them as signal fragments for the query plasmid. We classify signal fragments into 8 bins based on the number of labs annotated to them. Among all the correctly predicted plasmids, around 57% have signal fragments with only one lab annotated (the true source lab) (Supplementary Figure 7).

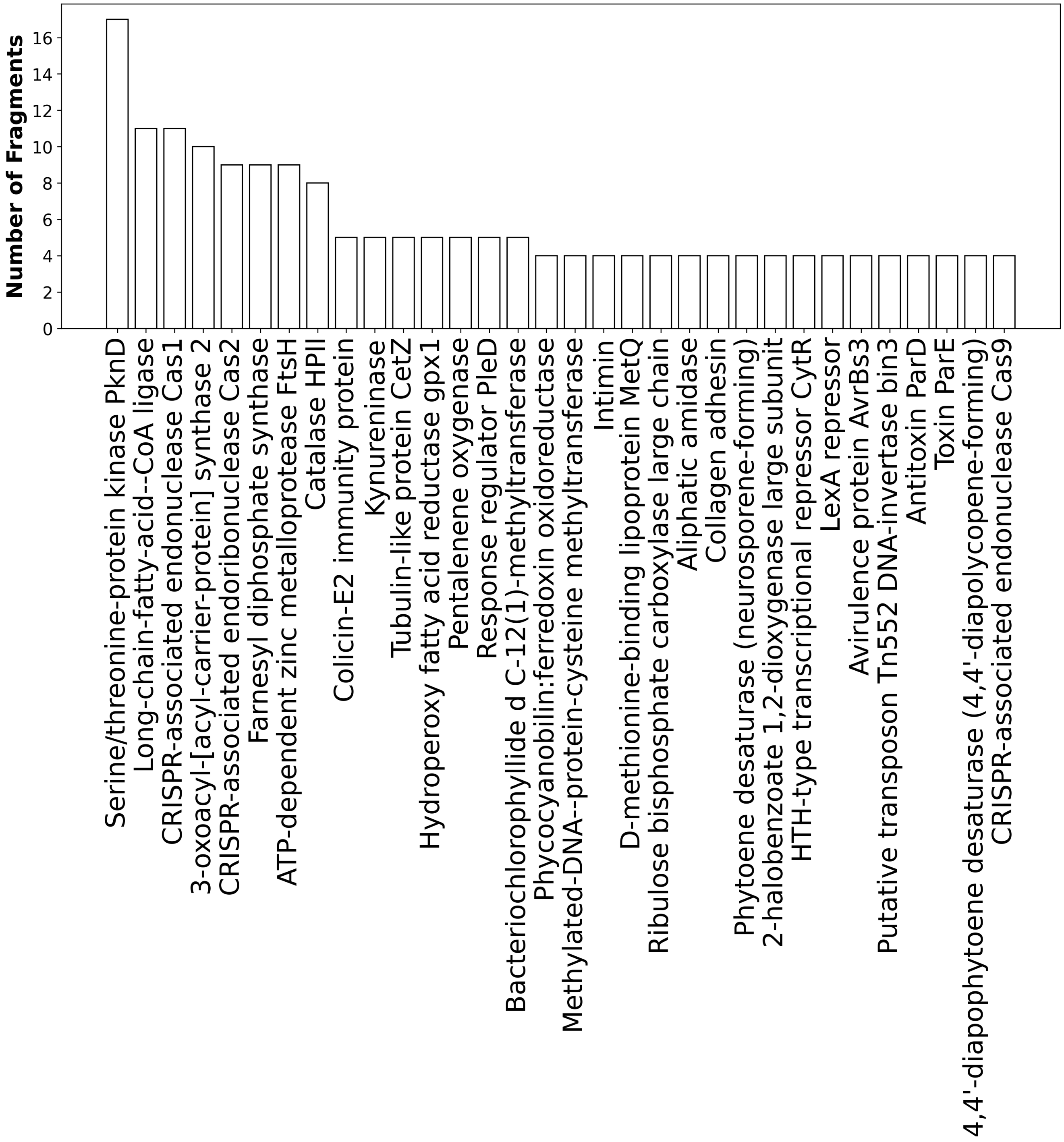

Supplementary Figure 8. Sequence functions that appear in at least four signal fragments across the five experiments.

Supplementary Note 4 The hypergeometric P-value used in this study has similar limitations as in the gene enrichment analysis case: 1) it neglects the dependence between fragments. Fragments next to each other in the plasmids are more likely to appear together. 2) Fragments do not all have equal chances to be selected. Fragments with large numbers of labs annotated to them are intrinsically more likely to be selected when compared with fragments with a small number of labs annotated to them. Despite these limitations, we believe the hypergeometric p-value is a reasonable representation for PlasmidHawk MAX mode significance. Gene enrichment analysis has come across similar problems as well. Although many studies have proposed numerous methods, such as Bayesian-based methods and probabilistic generative models, to overcome these limitations, there is no standard p-value calculation in gene enrichment study other than the hypergeometric p-value. Therefore, we believe, the hypergeometric p-value is a reasonable approach in calculating the significance of predicted labs returned by PlasmidHawk MAX mode. While the p-value could likely be further refined to include a wide array of further confounding factors (such as fragment dependence mentioned above), this is a non-trivial process and is left as an open research question.

In particular, the hypergeometric p-value is useful when a handful of labs occupy a large majority of the pan-genome fragments. In this case, the large p-value signifies that labs returned by PlasmidHawk MAX mode are likely by chance. For example, in the case where the pan-genome has 1000 fragments and lab A has 999 out of 1000 fragments in the pangenome. In this situation, for any given query sequence, PlasmidHawk MAX mode is likely to return lab A as the predicted lab. Despite this, we do not suggest users use p-values as the only indicator to decide whether the predictions are accurate or not. The p-value should serve as a general guidance and not the final deciding factor.
