# Supplementary material for: PlasmidHawk improves lab of origin prediction of engineered plasmids using sequence alignment
Source: Nat Commun. 2021 Feb 26;12:1167. doi: 10.1038/s41467-021-21180-w (PMC7910462; doi:10.1038/s41467-021-21180-w)
Supplement: Supplementary file 2 — Reporting Summary [file 41467_2021_21180_MOESM2_ESM.pdf]

nature research

Corresponding author(s): Todd J Treangen

Last updated by author(s): Dec 30, 2020

Reporting Summary

Nature Research wishes to improve the reproducibility of the work that we publish. This form provides structure for consistency and transparency in reporting. For further information on Nature Research policies, see our Editorial Policies and the Editorial Policy Checklist.

Statistics

For all statistical analyses, confirm that the following items are present in the figure legend, table legend, main text, or Methods section.

n/a	Confirmed	
☐	☒	The exact sample size (n) for each experimental group/condition, given as a discrete number and unit of measurement
☐	☒	A statement on whether measurements were taken from distinct samples or whether the same sample was measured repeatedly
☐	☒	The statistical test(s) used AND whether they are one- or two-sided<br>*Only common tests should be described solely by name; describe more complex techniques in the Methods section.*
☐	☒	A description of all covariates tested
☐	☒	A description of any assumptions or corrections, such as tests of normality and adjustment for multiple comparisons
☐	☒	A full description of the statistical parameters including central tendency (e.g. means) or other basic estimates (e.g. regression coefficient) AND variation (e.g. standard deviation) or associated estimates of uncertainty (e.g. confidence intervals)
☐	☒	For null hypothesis testing, the test statistic (e.g. F, t, r) with confidence intervals, effect sizes, degrees of freedom and P value noted<br>*Give P values as exact values whenever suitable.*
☒	☐	For Bayesian analysis, information on the choice of priors and Markov chain Monte Carlo settings
☐	☒	For hierarchical and complex designs, identification of the appropriate level for tests and full reporting of outcomes
☐	☒	Estimates of effect sizes (e.g. Cohen's d, Pearson's r), indicating how they were calculated

Our web collection on statistics for biologists contains articles on many of the points above.

Software and code

Policy information about availability of computer code

Data collection	Raw sequences are available from Addgene. No software was used to collect the data
Data analysis	PlasmidHawk is written in Python and is available at https://gitlab.com/treangenlab/plasmidhawk.git. All the scripts, including p-value calculation, used in this study to generate results and figures are available at https://gitlab.com/treangenlab/plasmidhawk/-/tree/data. The DOI of the repository is https://doi.org/10.5281/zenodo.4405001<br>scipy.stats.hypergeom is 1.4.1. sklearn version is 0.22.2.post1.

For manuscripts utilizing custom algorithms or software that are central to the research but not yet described in published literature, software must be made available to editors and reviewers. We strongly encourage code deposition in a community repository (e.g. GitHub). See the Nature Research guidelines for submitting code & software for further information.

Data

Policy information about availability of data

All manuscripts must include a data availability statement. This statement should provide the following information, where applicable:

- Accession codes, unique identifiers, or web links for publicly available datasets
- A list of figures that have associated raw data
- A description of any restrictions on data availability

TThe commands used and the source code are available on GitLab. Due to data sharing constraints, we are not permitted to redistribute the plasmid DNA sequences deposited in AddGene's repository. The plasmid sequences are available individually from the AddGene website (https://www.addgene.org/browse/) for download, and available for bulk download from AddGene upon request. Intermediate data is available by request and all of the methods, experimental results and scripts open source and available on GitLab (https://gitlab.com/treangenlab/plasmidhawk.git). The DOI of the repository is https://doi.org/10.5281/zenodo.4405001. The pCI-YFP plasmid is available from Genbank via Accession JQ394803.1

Field-specific reporting

Please select the one below that is the best fit for your research. If you are not sure, read the appropriate sections before making your selection.

☒ Life sciences ☐ Behavioural & social sciences ☐ Ecological, evolutionary & environmental sciences

For a reference copy of the document with all sections, see nature.com/documents/nr-reporting-summary-flat.pdf

Life sciences study design

All studies must disclose on these points even when the disclosure is negative.

Sample size	In each experiment, a total of 38,682 full-length plasmids from 896 labs are used in this study. We use all the plasmids downloaded from Addgene except plasmids from labs which have less than 10 plasmids in their labs. The reason of excluding those plasmids is that the deep learning method we compare in the paper excludes those plasmids, as the machine learning method need certain amount of plasmids from each lab to train the neural network in order to have a better comparison. Therefore, to have a fair comparison, we also exclude those plasmids in our study.
Data exclusions	We only used plasmids from labs, which have at least 10 plasmids in their labs. The reason of excluding those plasmids is that the deep learning method we compare in the paper excludes those plasmids, as the machine learning method need certain amount of plasmids from each lab to train the neural network in order to have a better comparison. Therefore, to have a fair comparison, we also exclude those plasmids in our study.
Replication	The benchmarking experiments have been repeated 5 times independently. All the replications are successful.
Randomization	In each experiment, 3 testing plasmids are selected randomly from each lab
Blinding	We were blinded during data collection and analysis.

Reporting for specific materials, systems and methods

We require information from authors about some types of materials, experimental systems and methods used in many studies. Here, indicate whether each material, system or method listed is relevant to your study. If you are not sure if a list item applies to your research, read the appropriate section before selecting a response.

Materials & experimental systems

n/a	Involved in the study	
☒	☐	Antibodies
☒	☐	Eukaryotic cell lines
☒	☐	Palaeontology and archaeology
☒	☐	Animals and other organisms
☒	☐	Human research participants
☒	☐	Clinical data
☒	☐	Dual use research of concern

Methods

n/a	Involved in the study	
☒	☐	ChIP-seq
☒	☐	Flow cytometry
☒	☐	MRI-based neuroimaging
